# AtLSG1-2 Regulates Leaf Growth by Affecting Cell Proliferation and the Onset of Endoreduplication and Synergistically Interacts with AtNMD3 during Cell Proliferation Process

**DOI:** 10.3389/fpls.2017.00337

**Published:** 2017-03-10

**Authors:** Huayan Zhao, Shiyou Lü, Liming Xiong

**Affiliations:** ^1^Applied Biotechnology Center, Wuhan Institute of BioengineeringWuhan, China; ^2^Division of Biological and Environmental Sciences and Engineering, King Abdullah University of Science and TechnologyThuwal, Saudi Arabia; ^3^Key Laboratory of Plant Germplasm Enhancement and Specialty Agriculture, Wuhan Botanical Garden, Chinese Academy of SciencesWuhan, China; ^4^Department of Horticulture Sciences, Texas A&M University, College StationTX, USA; ^5^Texas A&M Agrilife Research Center, DallasTX, USA

**Keywords:** AtLSG1-2, leaf growth, cell division, endoreduplication, AtNMD3

## Abstract

AtLSG1-2 is a circularly permuted GTPase required for ribosome biogenesis and recently shown to be involved in early leaf development, although it was unclear how AtLSG1-2 affects leaf growth. Here, we found that *atlsg1-2* mutants had reduced leaf size as a result of decreased cell size and cell number. Leaf kinematic analysis and CYCB1;1::GUS expression pattern in *atlsg1-2* mutant indicated that loss of function of *AtLSG1-2* delays the transition from cell division to cell expansion. Decreases in ploidy levels and trichome branch number suggest that AtLSG1-2 deficiency suppresses endoreduplication. Real-time PCR analysis showed that genes specifically expressed in the proliferation stage were highly expressed and those involved in endoreduplication were differentially regulated. LSG1 is known to mediate the recruitment of nucleocytoplasmic shuttling protein NMD3 back to the nucleus in yeast, yet their relationship was unclear in plants. Our genetic analysis revealed that the *atlsg1 atnmd3* double mutant displayed enhanced phenotypes as compared with the respective single mutant and that AtLSG1-2 and AtNMD3 synergistically affect the cell proliferation process.

## Introduction

The leaves of higher plants are important structures where photosynthesis takes place that provides carbon and energy for plant growth. Leaf development is a complicated process that is coordinately regulated by internal factors and environmental conditions. The final size of a leaf is determined by two factors: cell size and cell number. Cell size is influenced by vacuolar volume, cell wall expansion, macromolecular synthesis in the cytoplasm and nuclei size (Marshall et al., 2012). Meanwhile, cell division controls cell number. Many genes that control cell size, cell number or both have been identified (Kessler and Sinha, 2004; Gonzalez et al., 2012; Weis et al., 2015). Genes controlling cell division include, for example, transcription factor genes, microRNAs, genes involved in hormone biosynthesis or signaling, cell-cycle-related genes, ribosome biogenesis, etc. On the other hand, genes involved in cell expansion are functionally related with cell wall formation, transcription, and DNA replication (Gonzalez et al., 2012; Weis et al., 2015).

Ribosome is the basic machine for protein production. Ribosome biogenesis and function are tightly linked to development in various species. Mutations in ribosome proteins (RP) genes cause either lethal effects or pleiotropic phenotypes (Weis et al., 2015). Certain RP genes involved in leaf development have been identified. Mutations in these genes affect either cell number or size or both. For instance, Arabidopsis *OLIGOCELLULA2(OLI2), OLI5*, and *OLI7* encode a yeast Nop2 homolog, RPL5A or RPL5B, respectively. Whereas these *oli* mutants display decreased cell numbers (Fujikura et al., 2009), leaves of the *rpl18c-1, rps21b-1*, and *rps28b-1* mutants are smaller than those of the wild-type because of their reduced cell areas. On the other hand, *rps6a-1* and *rps6a-3* mutants showed strong reductions both in the cell size and in cell number in leaves (Horiguchi et al., 2011). The deficiency of three ribosome biogenesis factors PESCADILLO, BLOCK OF PROLIFERATION1, and WD REPEAT DOMAIN12 inhibits cell-cycle progression, which results in the defective cell growth and proliferation (Ahn et al., 2016). These studies point to an important role of ribosomal proteins in leaf development, although the mechanisms are still under investigation.

LSG1 is a circularly permuted GTPase whose function has been well studied in yeast. The nucleocytoplasmic shuttling protein NMD3 is an adaptor for the export of the large ribosomal subunit (60S) from the nucleus. LSG1 appears to recycle NMD3 from the cytosol to the nucleus and its deficiency causes an accumulation of NMD3 in the cytoplasm and indirectly affects the export of the large ribosomal subunit (60S) from the nucleus (Hedges et al., 2005). In humans, its ortholog HLsg1 is essential for cell growth and it shuttles between the nucleus and the cytoplasm (Reynaud et al., 2005). In Drosophila, the ortholog of Lsg1 Nucleostemin 3 (NS3) is essential for ribosome production and autonomous cell growth. Overexpression of *NS3* in yeast *lsg1* mutants partially rescues this lethal mutant, suggesting that it conserves functions in ribosome biogenesis (Hartl et al., 2013).

Whereas yeast, human, and Drosophila only have one copy of the *LSG1* gene, Arabidopsis has two copies, AtLSG1-1 and AtLSG1-2. The protein sequences of the two LSG genes share high identity, suggesting their functional redundancy. Expression analysis with fluorescent fusion proteins showed that the two proteins are cytosolic (Zhao et al., 2015), similar to their yeast orthologs. Results from our work (Zhao et al., 2015) and those of a recent study showed that the expression of AtLSG1-1 or AtLSG1-2 can partially rescue the yeast *lsg1* mutant (Weis et al., 2014; Zhao et al., 2015), suggesting that the two proteins share similar functions as their yeast orthologs. In plants, AtLSG1-2 appears to play a dominant role since the *atlsg1-2* null mutant showed pleiotropic phenotypes, including small size, short roots, delayed lateral root emergence, and distorted auxin homeostasis (Zhao et al., 2015), whereas AtLSG1-1 deficiency only subtly effected plant development (Weis et al., 2014). Furthermore, the expression level of *AtLSG1-2* is higher than that of *AtLSG1-1* (Zhao et al., 2015).

In this study, we focus on the roles of AtLSG1-2 on leaf development and investigated how loss of function of AtLSG1-2 may affect leaf growth. Compared to wild-type plant, *atlsg1-2* mutant had reduced leaf size. Leaf kinematic analysis and flow cytometry analysis revealed that cell division, differentiation and endoreduplication processes were obviously affected in *atlsg1-2* mutant. We also investigate the relationship between LSG1 and NMD3 in plants by exploring their genetic interactions between AtLSG1-2 and AtNMD3.

## Materials and Methods

### Plant Material and Growth Conditions

T-DNA insertion lines (*atlsg1-2*: Salk_114083 and *atnmd3*: WiscDsLox257G09) were obtained from Arabidopsis Biological Research Center. Wild-type (Accession Columbia-0) and mutant seeds were surface-sterilized in 50% bleach solution for 5 min and rinsed with water five times. The sterilized seeds were sown on agar-solidified half-strength Murashige and Skoog medium and incubated at 4°C for 3 days before being transferred to a growth chamber at 22°C with a 16-h-light/8-h-dark photoperiod. For soil-grown plants, 12-day-old seedlings growing on a petri dish were transferred to soil and grown in a growth room at 22°C with a 16-h-light/8-h-dark photoperiod.

For T-DNA line WiscDsLox257G09, the mutant allele was verified by PCR genotyping and by sequencing the PCR products. T-DNA insertion was detected using the left border primer (LB) combined with gene- specific primers LP and RP. Primer sequences are given in **Supplementary Table S2**.

### Phenotype Analysis

Leaf size, cell number, and size of abaxial epidermis were measured in the fifth leaf of 4-week-old plants growing in soil. A small droplet of superglue was applied on the glass slide. The leaf abaxial side was tightly imprinted on the slide coated with superglue for 30 s and the leaf was then quickly removed. The imprinted slides were observed and photographed with a microscope (Axio Imager Z2). Data from 5 to 8 leaves were used for statistical analysis.

### Plasmid Construction and Plant Transformation

To generate promoter deletion fusion constructs, different promoter deletions were cloned into the pENTRTM/D-TOPO vector using pENTRTM directional TOPO^®^ cloning kit (Invitrogen) and then subcloned into the binary vector pMDC162 by the LR recombination reaction. The plasmids were transferred into *Agrobacterium tumefaciens* and Arabidopsis plants were transformed by the floral dipping method (Clough and Bent, 1998).

### Leaf Kinematic Analysis

Leaf kinematic analysis was performed essentially as described by De veylder et al. (2001) with some modifications. Leaves were submerged in 10 μM FM4-64 for 10 min and destained in water. The epidermal cells of stained leaf abaxial side were observed and photographed with a microscope (Axio Imager Z2). The experiment was repeated three times with similar results obtained. Results from only one set of the experiment were presented in this study.

### GUS Staining

Whole seedlings were incubated in ice-cold 90% acetone for 2 h, washed in 100 mM Na_3_PO_4_ (pH 7.0) and subsequently immersed in 5-bromo-4-chloro-3-indolyl-β-D-glucuronide (X-Gluc) buffer (100 mM Na_3_PO_4_ buffer, pH 7.0, 10 mM Tris, pH 8.0, 1 mM EDTA, 0.05% Triton X-100, 1 mg/ml X-Gluc) at 37°C overnight. Chlorophyll was cleared in 70% ethanol. The cleared samples were photographed with a Nikon SMZ25 stereomicroscope.

### Flow Cytometry Analysis

Preparation of plant materials for flow cytometric assays was performed as described in Dolezel et al. (2007). In brief, leaves were quickly chopped with a razor blade in ice-cold Galbraith’s buffer [45 mM MgCl_2_, 20 mM MOPS, 30 mM sodium citrate, and 0.1% (vol/vol) Triton X-100, pH 7.0]. The homogenate was filtered through 70 μM nylon mesh. Fifty microgram per milliliter propidium iodide (PI) combined with 50 μg/ml of RNase A was added into the filtered homogenate and mixed for flow cytometry analysis. The sample was analyzed in a BD LSRFortessa flow cytometer equipped with a 50 mW 561 nm laser.

### RNA Sequencing and Quantitative Real-Time PCR

Total RNA was extracted from the first pair of 11-day-old wild-type plant and 13-day-old mutant leaves using an RNasy Plant Mini Kit (Qiagen). Residual DNA was removed with DNase I (NEB) and 2 μg of cleaned RNA were used for reverse transcription. RNA sequencing was performed according to Chen et al. (2013). For real-time PCR, reverse transcription was conducted using the SuperScript III first-strand synthesis SuperMix (Invitrogen) and PCR was conducted using Power SYBRgreen PCR Master Mix (Applied Biosystems) in a 7900 HT Fast Real-Time PCR System (Applied Biosystems). *ACTIN2* was used as the internal control. Three biological replicates were performed for real-time PCR analysis. For checking the transcript level of *AtNMD3* in the T-DNA insertion line WiscDsLox257G09, RNA was extracted from the wild-type plant and the mutant seedlings and real-time PCR was performed as described above.

## Results

### Small-Sized *atlsg1-2* Leaves Are Caused by Reduced Cell Size and Cell Number

**FIGURE 1:**
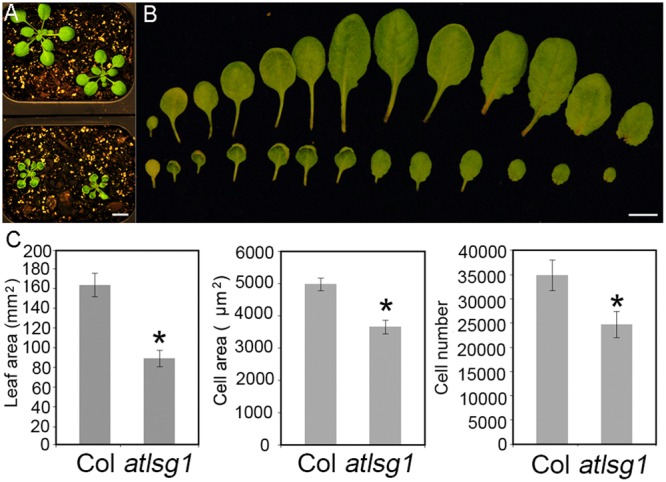
**The *atlsg1-2* mutant has smaller leaf size, cell number and cell size. (A)** Morphology of 4-week-old wild-type (upper panel) and *atlsg1-2* mutants (lower panel) growing in soil. Scale bars indicate 1 cm. **(B)** Morphology of detached leaves of the wild-type (upper panel) and *atlsg1-2* mutants (lower panel). Cotyledon and leaves 1–12 are arranged from left to right. Scale bars indicate 1 cm. **(C)** Statistical analysis of leaf area, cell size and cell number of the fifth leaf. Data are means and standard derivations (*n* = 5, ^∗^*P* < 0.01 by Student’s *t*-test).

In a genetic screen for mutants defective in lateral root response to drought and abscisic acid, we isolated a mutant, *dig6* (*d*rought *i*nhibited *g*rowth of lateral roots), which showed reduced lateral root numbers. Map-based cloning identified that the mutation occurred in the *AtLSG1-2* gene (Zhao et al., 2015). The *dig6* mutant and a T-DNA insertion mutant allele *atlsg1-2* (Salk_114083) had nearly identical phenotypes (Zhao et al., 2015). Here, we focused on the T-DNA insertion mutant *atlsg1-2*. Young leaves of the mutant exhibited significantly retarded growth and mature leaves remained small (**Figures 1A,B**). By checking the fifth leaves of the wild-type and *atlsg1-2* mutants in detail, we found that the leaf area of the mutant was half that of the wild-type, as shown in **Figure 1C**. To determine which factor contributed to leaf size reduction, we evaluated cell size and cell number and found that in the mutant, cell size and cell number decreased to 73.5 and 70.9% that of the wild-type plant, respectively.

### Leaf Kinematic Analysis

**FIGURE 2:**
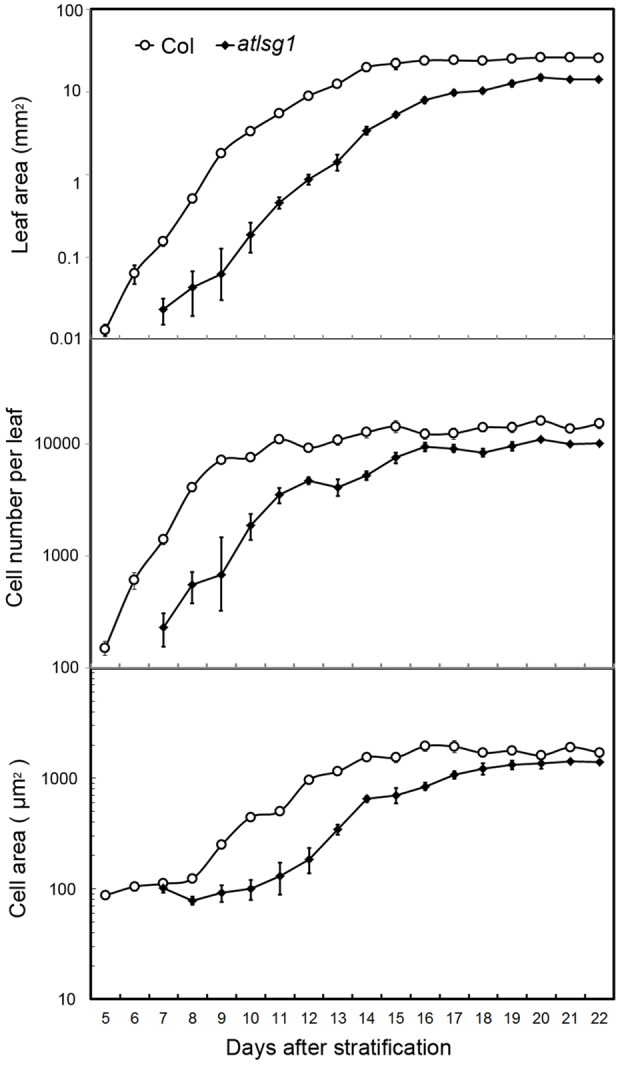
**Kinematic analysis of the first pair of leaves.** The first pair of leaves was collected daily from the wild-type and mutants. At least five samples were used for statistical analyses. The experiment was repeated three times with similar results obtained.

Cell size and cell number are closely associated with cell expansion and cell proliferation activity, respectively. We conducted a leaf kinematic analysis to investigate how loss of function of *AtLSG1-2* affects cell division and expansion. Leaf development is divided into three stages: cell division, expansion, and maturation (Beemster et al., 2005). We evaluated leaf development in the first pair of *atlsg1-2* leaves. As shown in **Figure 2**, compared to the wild-type, leaf emergence was delayed by 2 days in the *atlsg1-2* mutant. Furthermore, leaf growth was relatively slower in the mutant and cell size and cell number also increased more slowly compared with the wild-type, suggesting that cell proliferation and expansion activity were simultaneously suppressed in the *atlsg1-2* mutant (**Figure 2**). Furthermore, the cell expansion phase was noticeably delayed in the *atlsg1-2* mutant (**Figure 2** and **Supplementary Figure S1**). In wild-type plants, cell size was approximately 100 μm^2^ 5–8 days after stratification (DAS), but after 9 DAS, cell size rapidly increased, suggesting that cell differentiation has begun. In the *atlsg1-2* mutant, cell size from 7 to 11 DAS was similar to that of the wild-type plant from 5 to 8 DAS, and although cell size increased after 11 DAS, the rate was apparently slower (**Figure 2**). As shown in **Supplementary Figure S1**, whereas most of the cells in 9-DAS-old wild-type leaves had already underwent expansion, those in the mutant leaves began to expand only at 13-DAS. This suggests that cell expansion activity was apparently impaired in the *atlsg1-2* mutant.

**FIGURE 3:**
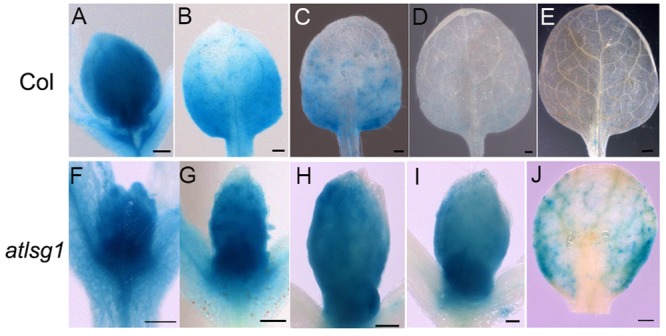
**Expression pattern of CYCB1;1::GUS in the wild-type and *atlsg1-2* mutant leaves.** The first pair of leaves was collected from 8 **(A,F)**, 10 **(B,G)**, 11 **(C,H)**, 12 **(D,I)**, and 14 **(E,J)** days after stratification. Six to ten plants were used for staining with similar results and the representative images are shown. Scale bars indicate 0.5 mm.

To substantiate the inhibitory effects of *AtLSG1-2* deficiency on the normal progression of cell division to differentiation, we examined the CYCB1;1::GUS activity in the wild-type and *atlsg1-2* mutants; *CYCB1;1* is specifically expressed in dividing cells (Ferreira et al., 1994). We found that GUS signals greatly diminished in 12-day-old wild-type leaves but were still strongly expressed in the 14-day-old mutant leaves (**Figure 3**). As shown in **Supplementary Figure S1**, many cells in 13-DAS-old mutants still underwent division whereas the cells in wild-type plant were expanded. This was consistent with *CYCB1;1* staining results. Therefore, loss of function of *AtLSG1-2* hindered the transition from cell division to expansion.

### AtLSG1-2 Deficiency Inhibits Endoreduplication

**FIGURE 4:**
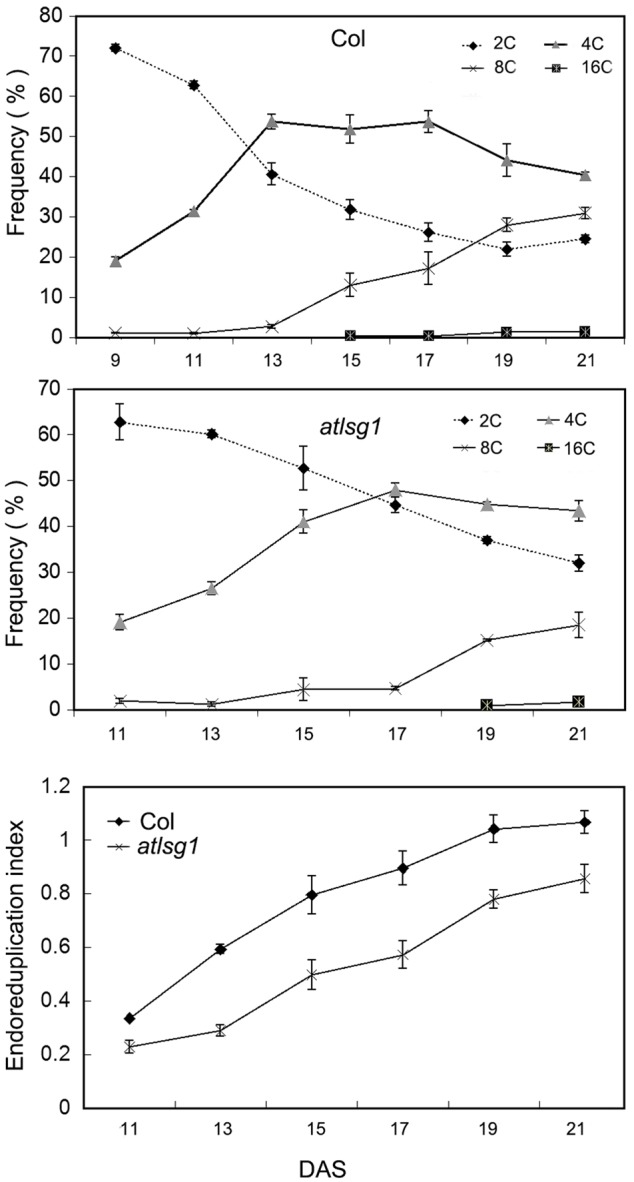
**Cytometry analysis of ploidy levels in the wild-type and *atlsg1-2* mutant.** The first pair of leaves was collected for flow cytometry analysis every 2 days starting from 11 days after stratification. The percentage of each ploidy type among total types was calculated. Endoreduplication index indicates the average number of endocycles undergone by a given nucleus. Data represent means and standard derivations from three biological replicates.

The leaf kinematic analysis showed that cell expansion processes were suppressed in the *atlsg1-2* mutant. Because cell expansion is closely related with the endoreduplication process, we examined the ploidy distribution by flow cytometry in the first pair of wild-type and *atlsg1-2* mutant leaves. Leaf samples were collected during cell division, cell expansion, and maturation phases. As shown in **Figure 4**, the 2C population decreased more slowly in *atlsg1-2* mutants than in wild-type leaves, while the 4C population slowly increased in the mutant. This suggests that cell expansion phase was delayed in the mutant. Although both 8C and 16C populations represent the onset of endoreduplication, the 16C population contributes minimally and thus will not be discussed here. The *atlsg1-2* mutant plants also had a more slowly increasing 8C population such that the final 8C frequency was around 30% in wild-type leaves but only about 20% in the *atlsg1-2* mutant (**Figure 4**), suggesting that the endoreduplication process was affected in the mutants. The ploidy distribution measured by flow cytometry can also be expressed as an endoreduplication index (EI), which represents the average number of endocycles undergone by a given nucleus. The EI, developmentally regulated during leaf growth, was lower in the *atlsg1-2* mutant throughout development, indicating that the average number of endocycles is reduced in the *atlsg1-2* mutant.

**FIGURE 5:**
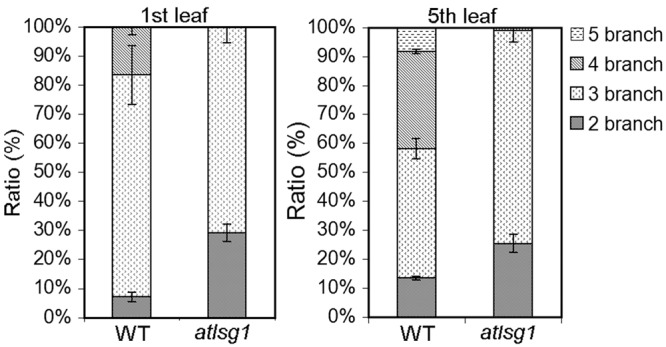
**The ratio of different types of trichomes in the wild-type and *atlsg1-2* mutant leaves.** Data are means and standard derivations from three biological replicates each with at least 5–8 leaves examined.

To verify these results, we also checked the ratio of trichomes with different numbers of branches because trichome branch number is positively correlated with ploidy level (Bramsiepe et al., 2010). In the first leaf, the ratio of two-branched trichome was significantly higher in *atlsg1-2* mutant leaves than in wild-type plants (**Figure 5**). In *atlsg1-2* mutants, most trichomes are two or three-branched, while wild-type plant leaves contain a relatively high ratio of four-branched trichomes and small number of five-branched trichomes (**Figure 5**). These results combined with those from flow cytometry suggest that an AtLSG1-2 deficiency suppresses endoreduplication.

### Genes Specifically Expressed in Proliferation Stage Are Upregulated in *atlsg1-2* Mutants and Genes Associated with Endoreduplication Were Differentially Regulated

**FIGURE 6:**
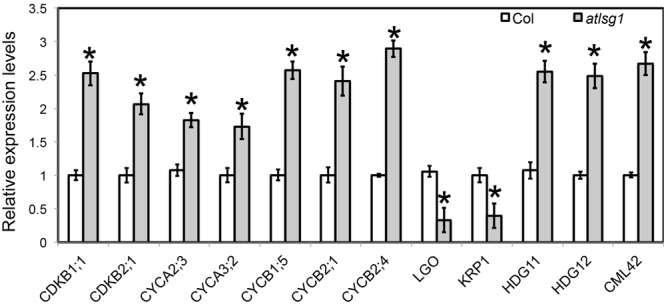
**Real-time PCR analysis of differentially expressed genes in the *atlsg1-2* mutant.** RNA was extracted from the first pair of leaves of 11-day-old wild-type and 13-day-old *atlsg1-2* mutant. *ACTIN2* was used as an internal control. Data are means and standard derivations (*n* = 3, ^∗^*P* < 0.01 by Student’s *t*-test). Error bars in the graph indicate standard derivations of three biological replicates for each gene.

Both leaf kinematic analysis and pCYCB1;1::GUS expression showed that the progression from cell division to differentiation was delayed in the *atlsg1-2* mutant, suggesting that the expression of those genes associated with this process might be differentially regulated. Because leaf emergence in the mutant was delayed 2 days compared with wild-type plants, to eliminate the time difference in leaf emergence, we chose the first pair of 11-day-old wild-type and 13-day-old *atlsg1-2* mutant leaves for RNAseq and qRT-PCR analysis. In 11-day-old wild-type plant leaves, small amount of cells in the tip of wild plant leaves expand and undergo differentiation. RNAseq results showed that cell-cycle process was obviously perturbed in the *atlsg1-2* mutant (**Supplementary Table S1**). We further checked the expression of those genes specifically expressed in proliferation stage (Beemster et al., 2005), which includes *CYCA2;3, CYCA3;2, CDKB2;1, CYCB1;5, CYCB2;1*, and *CYCB2;4*, etc. Most of these genes were highly expressed in *atlsg1-2* mutant (**Supplementary Table S1**). While the cause and effect relation of these events is unclear, one possibility is that *AtLSG1* deficiency probably affects ribosome biogenesis and compromises the capacity of protein synthesis. The insufficient ability for protein translation may cause slower proliferation and delayed cell-cycle exit, which resulted in higher expression of cell-cycle genes later in proliferation stage in the mutant. Since RNA-seq analysis was only performed once, the expression patterns of some genes specifically expressed in proliferation stage were further confirmed by using real-time PCR (**Figure 6**). It could be concluded that the prolonged cell division phase in the *atlsg1* mutant correlated with high levels of these genes specifically expressed in proliferation stage.

Leaf kinematic analysis showed that the onset of endoreduplication was affected in the *atlsg1-2* mutant, suggesting that the expression of some genes associated with this process might be altered. Our RNA-seq analysis (**Supplementary Table S1**) showed that several key regulators of endoreduplication including *KRP1, CYCA2;3, CDKB1;1*, and *LGO* were differentially regulated in *atlsg1-2* mutants. Among these genes, *KRP1* and *LGO* are positive regulators of endoreduplication. *KRP1* encodes a cyclin-dependent kinase inhibitor protein that negatively regulates cell division and promotes endoreduplication (Weinl et al., 2005). *LGO* is a member of a plant-specific cell-cycle-inhibitor-family SIAMESE and is essential for endoreduplication of sepal giant cells. Its loss has caused the absence of giant cells in leaves and sepals (Roeder et al., 2010). RNA-seq data showed that the transcript levels of *KRP1* and *LGO* were reduced considerably in *atlsg1-2* mutants. On the other hand, the expression of *CDKB1;1* and *CYCA2;3*, which encode negative regulators of endoreduplication, significantly increased in the mutant (**Supplementary Table S1**). *CYCA2;3*, encoding A-type cyclin, could form a functional complex with CDKB1;1 and suppress the onset of endoreduplication (Boudolf et al., 2009). Other genes associated with endoreduplication were also differentially expressed in the mutant (**Supplementary Table S1**); these genes were associated with signaling pathways or transcription factors. For example, *CALMODULIN LIKE 42* (*CML42*), a calmodulin-related calcium sensor: a *cml42* knockout mutant had abnormal trichomes with increased branching (Dobney et al., 2009), suggesting that it is a negative regulator of trichome branching. Its expression was significantly upregulated in the *atlsg1-2* mutant. HOMEODOMAIN GLABROUS 11 and 12, belonging to the HD-ZIP IV family, also negative regulators of trichome branching (Nakamura et al., 2006), had upregulated expression levels in the *atlsg1-2* mutant. These results from RNA-seq analysis suggest that the expression of these genes might be affected in the mutant. We thus conducted real-time PCR to validate and quantify the expression level of these genes. Our real-time PCR results are consistent with those of the RNAseq analysis (**Figure 6**). Therefore, the differential expression of these endoreduplication regulatory genes may lead to disturbed endoreduplication as observed in the mutant.

### Simultaneous Knockout of *AtLSG1-2* and *AtNMD3* Exhibits Synergistic Effects

**FIGURE 7:**
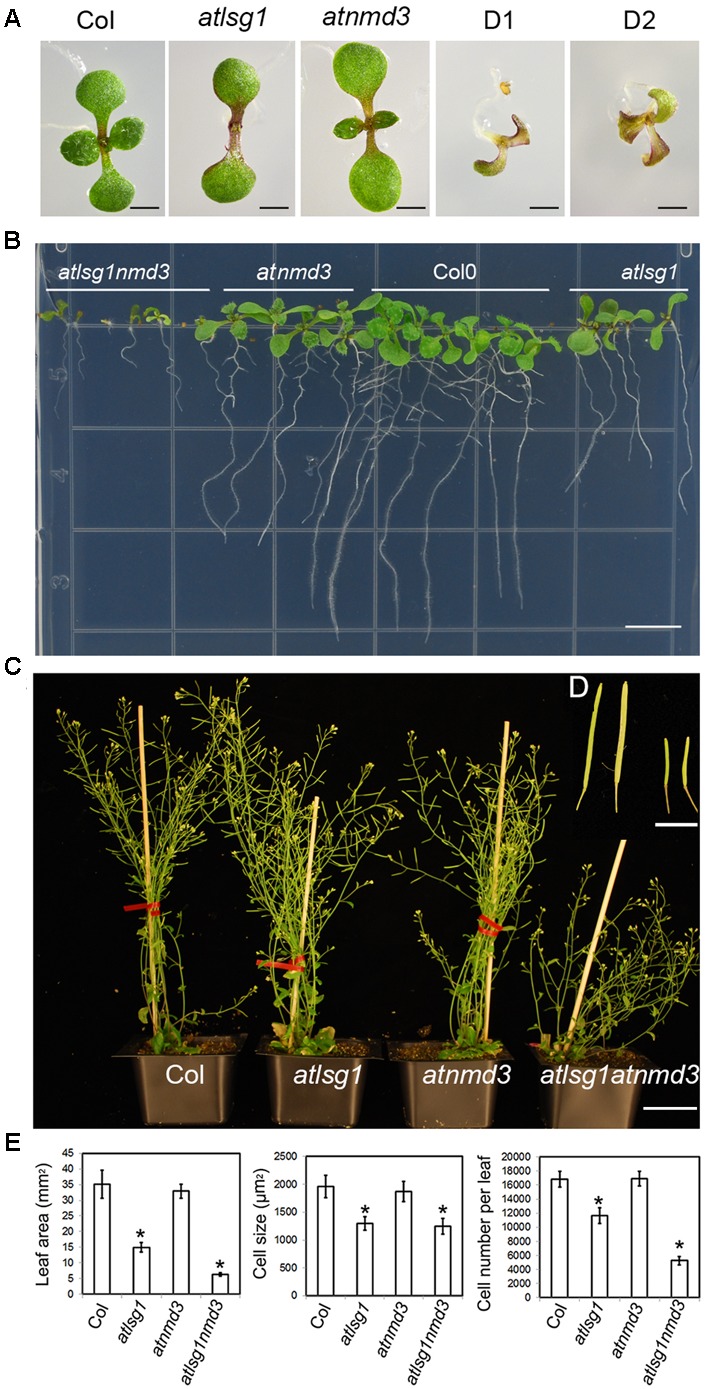
**Phenotypes of *atlsg1, atnmd3*, and *atlsg1 atnmd3* mutants. (A,B)** Morphology of 11-day-old seedlings growing on horizontally **(A)** and vertically **(B)** placed MS agar plates. The *atlsg1 atnmd3* double mutant has two (D1) or three cotyledons (D2). **(C)** Morphology of 44-day-old plants growing in soil. Bars in **(A,B)** represent 0.5 and 1 mm, respectively. **(D)** (insert) A close up of siliques. From left to right the plants are wild-type, single-mutant *atlsg1*, single-mutant *atnmd3*, and double-mutant *atlsg1 atnmd3*. The scale bar represents 0.5 cm. **(E)** Leaf phenotype of single and double mutants. Leaf area, cell size and cell number were examined in fifth leaves of 4-week-old plants. At least five plants each genotype was used for statistical analysis.

LSG1 is known to mediate the export of the ribosome export factor NMD3 from the nucleus in yeast, but how plant NMD3 homologs interplay with LSG1 remains unknown. To understand the genetic interaction between AtLSG1-2 and AtNMD3 in Arabidopsis, we obtained an *atnmd3* knockdown mutant (WiscDsLox257G09) because a *atnmd3* knockout mutant is lethal (Chen et al., 2012). In the *atnmd3* knockdown mutant, a T-DNA fragment was inserted in its 3′-untranslated region. The expression level of the *AtNMD3* transcripts in the homozygous line was examined by RT-PCR using gene-specific primers (**Supplementary Figure S2** and **Table S2**) and was found significantly reduced (**Supplementary Figure S2**). Nonetheless, this mutant showed only subtle phenotype changes at a very early stage such as retarded growth, delayed leaf emergence, and stunted roots (**Figure 7**). We generated an *atlsg1 atnmd3* double mutant by genetic crossing of the two single mutants and examined the phenotype of the resulting double mutant. The growth of the primary root of the double mutant was very slow and leaf emergence was also significantly delayed in the early seedling stage (**Figures 7A,B**). The stature of the adult double mutant was much shorter than either single mutant (**Figure 7C**). The dwarf and bushy phenotypes along with short siliques and low fertility (**Figures 7C,D**) were not seen in either single mutant. We thus conclude that the attenuation of *AtNMD3* function enhances the phenotypes caused by *AtLSG1-2* loss. We examined the phenotypes of the first pair of leaves in more details. As shown in **Figure 7E** and **Supplementary Figure S3**, *atlsg1-2* leaf area was reduced, whereas that of the *atnmd3* mutant was similar to that of the wild-type. The leaf area of the double mutant was considerably less than either single mutant. Although cell size of the double mutant was similar to that of the *atlsg1-2* mutant, cell number was significantly reduced compared with either single mutant (**Figure 7E**). These results suggest that AtLSG1-2 and AtNMD3 synergistically control cell proliferation activity during leaf development.

## Discussion

The functions of LSG1 proteins have been studied in yeasts, humans, Drosophila, and now in plants. Despite their different subcellular localization patterns, their roles in ribosome biogenesis have consistently been confirmed. Moreover, the complete loss of these genes is usually lethal, suggesting that the ribosome biogenesis processes they are involved in are essential for cell viability. The identification of weak alleles makes it feasible to investigate the roles of these genes in growth and development. *ns3* is a knockdown fly mutant with a P-element insertion in the *NS3* gene (Kaplan et al., 2008) that results in small body size because fewer and smaller cells are produced. However, further study showed that these phenotypic defects could be rescued by the expression of *AKT1*, a central effector of the insulin-signaling pathway, whose activation affects a number of downstream effectors to stimulate ribosome biogenesis (Kaplan et al., 2008). Therefore, NS3-mediated body size acts through the insulin-signaling pathway. In Arabidopsis, there are two LSG1 paralogs. Because of their conserved functions in ribosome biogenesis and functional redundancy, loss of either gene only mildly affects plant growth (Weis et al., 2014; Zhao et al., 2015). Nonetheless, whereas the loss of AtLSG1-1 had little effect on plant growth, *atlsg1-2* mutants showed pleiotropic phenotypes, suggesting that AtLSG1-2 has more important roles. A recent study identified that the *AtLSG1-2* gene is involved in the 40S ribosome maturing process (Weis et al., 2014). Our study showed that AtLSG1-2 loss of function caused the decreased levels of 40S, 60S, and 80S ribosomes (Zhao et al., 2015), demonstrating its importance in ribosome biogenesis. Furthermore, defective ribosome biogenesis seems to be closely related with the phenotypes observed in *atlsg1-2* mutants; some similar phenotypes were shown in other mutants with defective ribosome biogenesis (Ito et al., 2000; Weijers et al., 2001). Because there is no insulin pathway in plants, AtLSG1-2 must act through different pathways to control leaf growth. Despite the possibility of different regulatory pathways of NS3 and AtLSG1, *NS3* and *AtLSG1-2* loss of function mutants share some common phenotypes: defective ribosome biogenesis, retarded growth and small size as a result of decreased cell size and reduced cell number, suggesting that NS3 and AtLSG1 share some conserved functions and that ribosome biogenesis which both genes are involved in is necessary for maintaining normal cell sizes. The various phenotypes caused by *AtLSG1-2* loss of function also overlapped with those reported for several RP gene mutants (Fujikura et al., 2009; Horiguchi et al., 2011; Weis et al., 2015). The reason for these phenotypes might be inefficient global protein synthesis, which impairs the cell-cycle progression and thus affects normal cell division and expansion activity. We also noticed that the *atlsg1-2* leaves displayed abnormal leaf polarity and auxin-defective phenotypes (Zhao et al., 2015). Thus it is also likely that dysfunctional ribosomes due to the lack specific ribosomal proteins may affect the translation of some specific mRNA involved in the leaf development process.

The interaction of LSG1 and NMD3 was well studied in yeast, but is still unknown in plants. In yeast, LSG1 participates in the nuclear export of NMD3 during ribosome biogenesis. The NMD3 ortholog in Arabidopsis was also demonstrated to be required for the nuclear export of 60S ribosomal subunit (Chen et al., 2012). Our current genetic analysis showed that *atlsg1 atnmd3* double mutant displayed smaller leaves compared to parental single mutants. Decreased leaf size is mainly associated with the further reduction of cell number in the double mutant (**Figure 7E**), suggesting that AtNMD3 may mainly affect cell division, which is different from AtLSG1-2 that controls both cell number and cell size. Specific roles by NMD3 in cell proliferation have also been demonstrated in rice, where overexpression of a dominant-negative form of truncated OsNMD3 led to a dwarf phenotype as a result of decreased cell number (Shi et al., 2014). These data suggest conserved functions of NMD3 in both monocotyledonous and dicotyledonous plants. The synergistic effects seen in the double mutant also implies that AtLSG1-2 and AtNMD3 act through common or shared pathways to regulate cell division, which is consistent with findings in other systems where the two proteins work together in ribosome biogenesis. Meanwhile, we found that AtLSG1-2 and AtNMD3 also have their own specific functions. *atlsg1-2* mutants displayed incurvate leaves and auxin-defective phenotypes (Zhao et al., 2015), which were not present in *atnmd3* mutants, suggesting unique roles by AtLSG1-2 in leaf polarity and auxin homeostasis. NMD3 proteins were found to be involved in secondary wall thickening and to control some agronomic traits including internode growth and panicle and seed development (Chen et al., 2012; Shi et al., 2014). These studies suggested that AtLSG1-2 might participate in the 60S subunit nuclear export mediated by AtNMD3, but that NMD3 may be also involved in other unknown pathways.

Primer sequence in this study was listed in **Supplementary Table S2**.

## Author Contributions

HZ designed, conducted the experiments, and wrote the manuscript. SL performed leaf kinematic analysis. LX supervised this work and revised the manuscript.

## Conflict of Interest Statement

The authors declare that the research was conducted in the absence of any commercial or financial relationships that could be construed as a potential conflict of interest.

## Supplementary Material

FIGURE S1 **Morphology of abaxial epidermal cells in the wild-type and *atlsg1* in different days after stratification (DAS).** Scale bars indicate 20 μm.

FIGURE S2 **Molecular characterization of the *atnmd3* mutant. (A)** The detection of T-DNA insertion by PCR analysis. Genomic DNA was extracted from wild-type and *atnmd3*. PCR was performed using the gene – specific primer LP and RP (lanes 1,3) or left border – specific primer (LB) and RP (lanes 2,4). **(B)** Transcript levels of *AtNMD3* in the wild-type and *atnmd3* mutant. RNA was extracted from the rosette leaves of the wild-type and the *atnmd3* mutant. Real-time PCR was preformed with primers OXH329 and OXH330. *ACTIN2* was used as internal control. Data are means and standard deviations (*n* = 3, ^∗^*P* < 0.01 by Student’s *t*-test).

FIGURE S3 **Leaf phenotype of 4-week-old plants. Scale bars indicate 1 cm**.

TABLE S1 **Differentially regulated genes**.

TABLE S2 **List of primers used in this study**.
