# Supplementary figures and images for: AtLSG1-2 Regulates Leaf Growth by Affecting Cell Proliferation and the Onset of Endoreduplication and Synergistically Interacts with AtNMD3 during Cell Proliferation Process
Source: Front Plant Sci. 2017 Mar 10;8:337. doi: 10.3389/fpls.2017.00337 (PMC5344897; doi:10.3389/fpls.2017.00337)

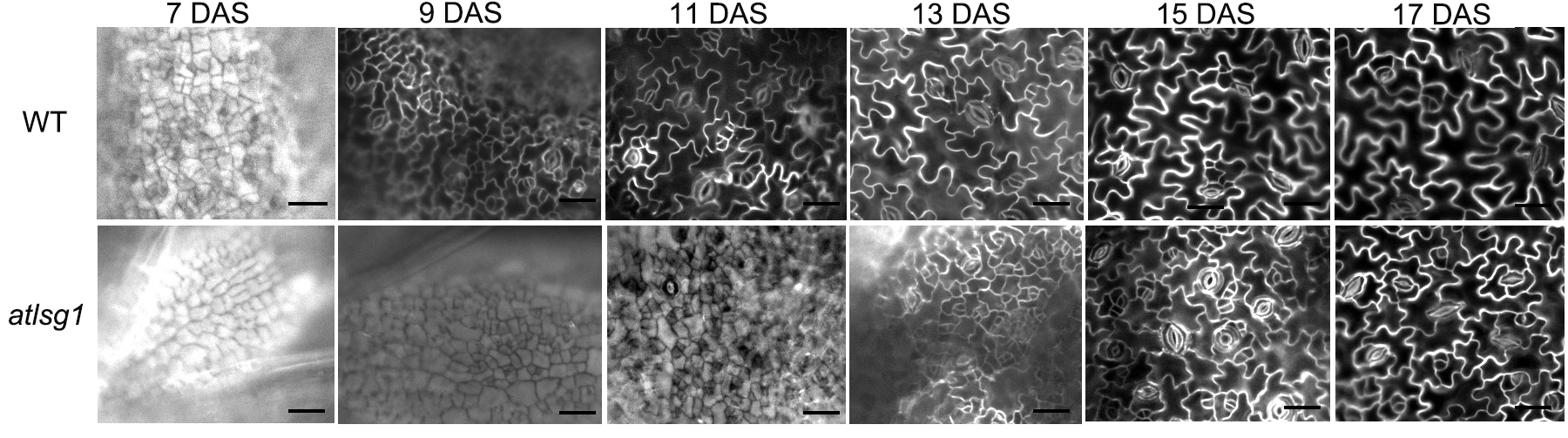

Supplement: FIGURE S1 — Morphology of abaxial epidermal cells in the wild-type and atlsg1 in different days after stratification (DAS). Scale bars indicate 20 μm. [file Image_1.JPEG]

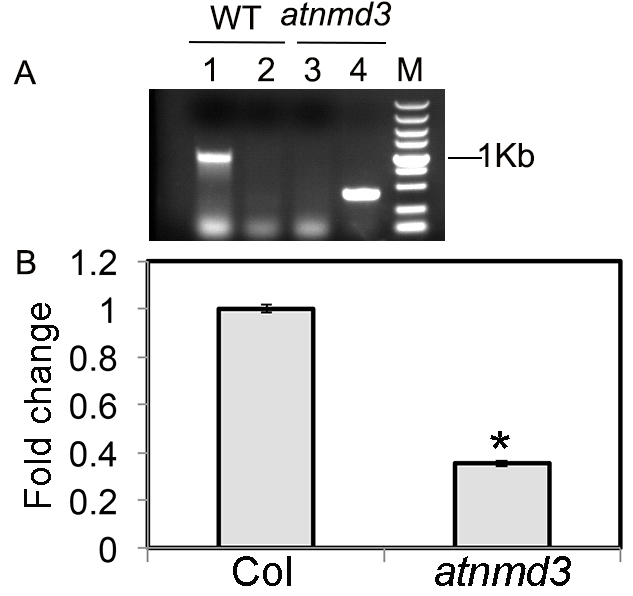

Supplement: FIGURE S2 — Molecular characterization of the atnmd3 mutant. (A) The detection of T-DNA insertion by PCR analysis. Genomic DNA was extracted from wild-type and atnmd3. PCR was performed using the gene – specific primer LP and RP (lanes 1,3) or left border – specific primer (LB) and RP (lanes 2,4). (B) Transcript levels of AtNMD3 in the wild-type and atnmd3 mutant. RNA was extracted from the rosette leaves of the wild-type and the atnmd3 mutant. Real-time PCR was preformed with primers OXH329 and OXH330. ACTIN2 was used as internal control. Data are means and standard deviations (n = 3, ∗P < 0.01 by Student’s t-test). [file Image_2.JPEG]

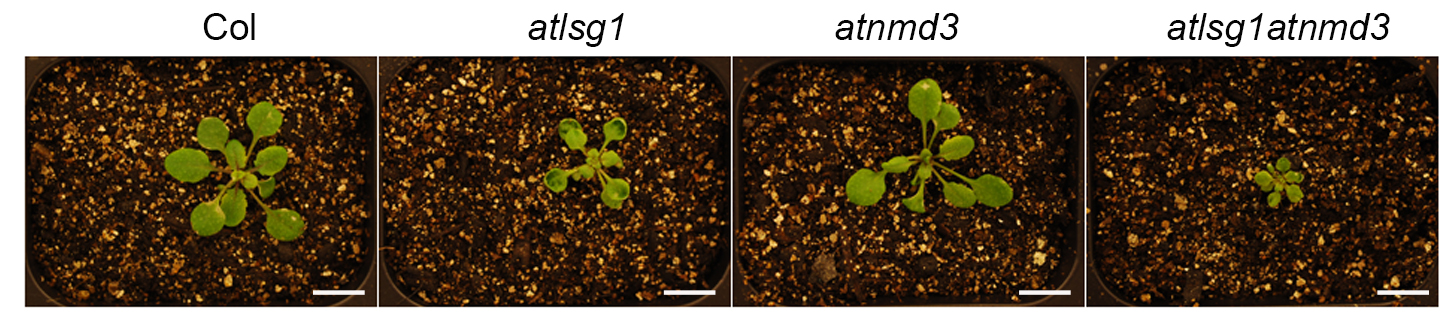

Supplement: FIGURE S3 — Leaf phenotype of 4-week-old plants. Scale bars indicate 1 cm. [file Image_3.JPEG]
